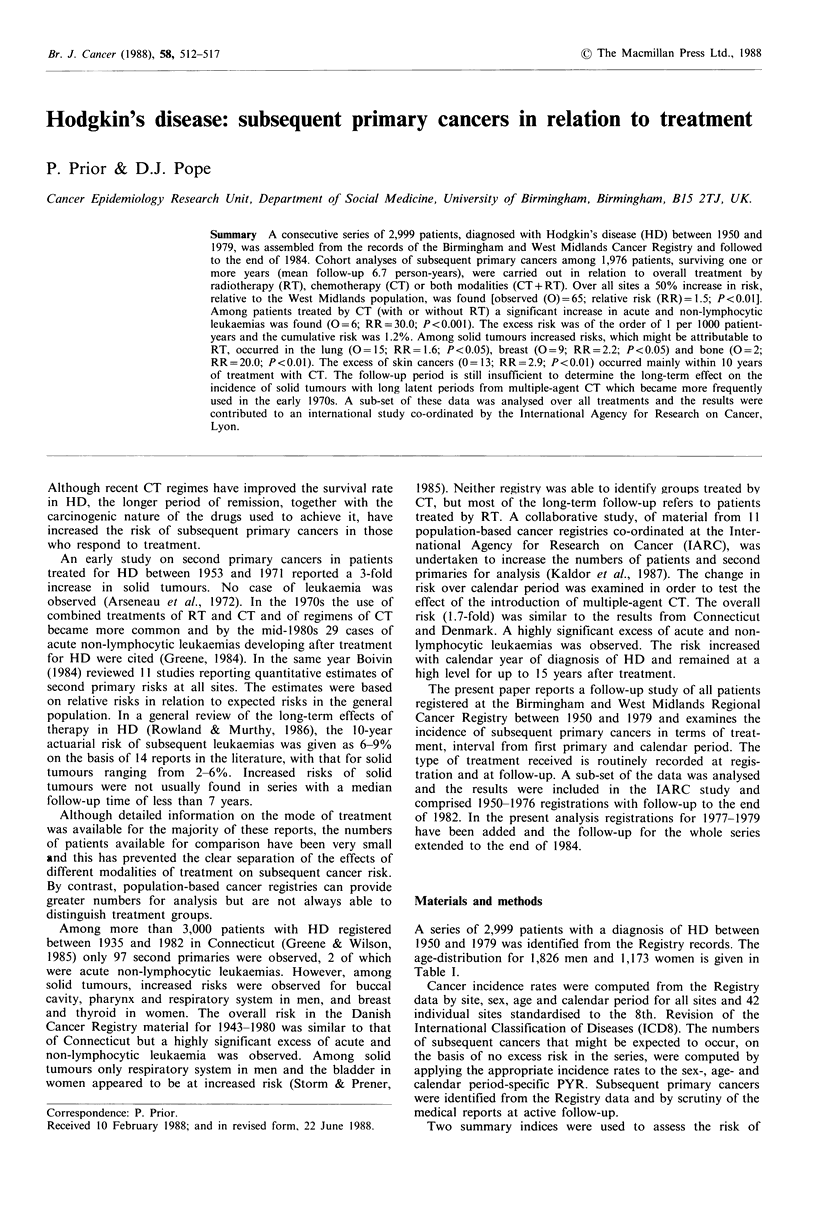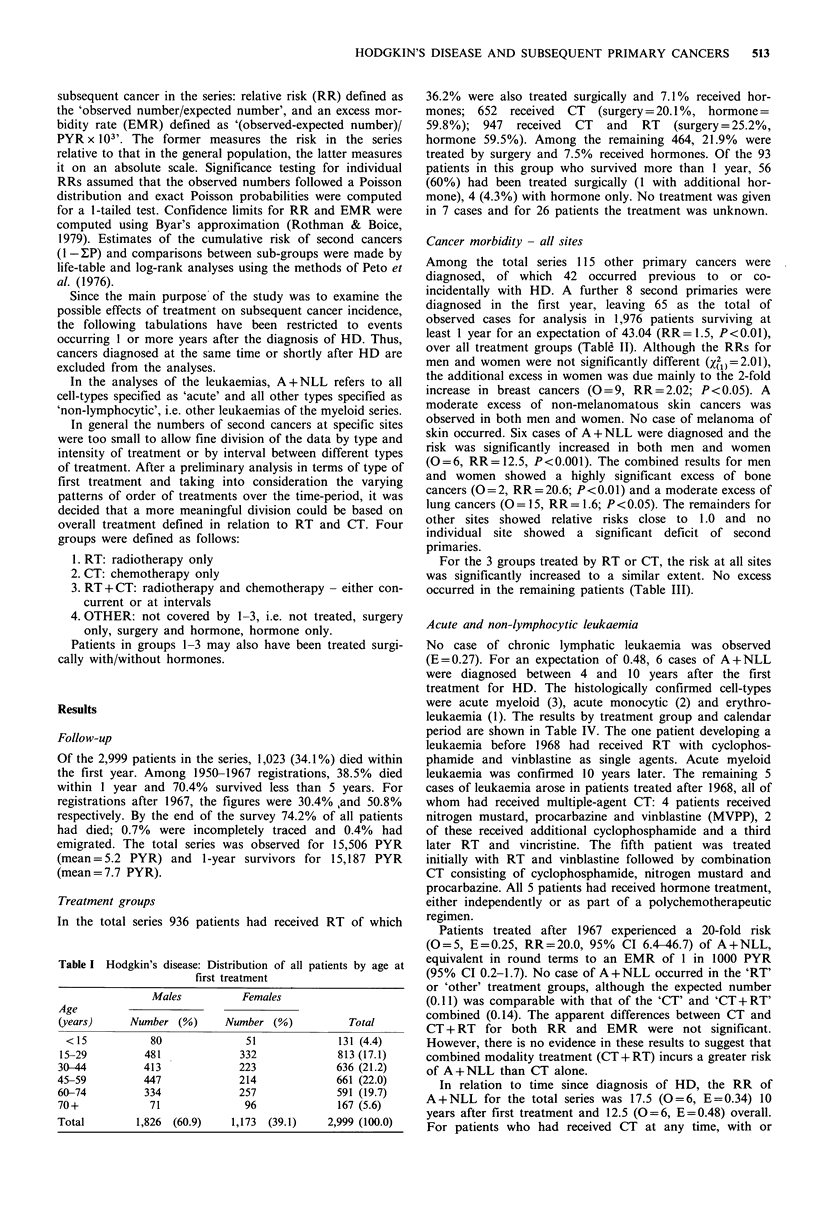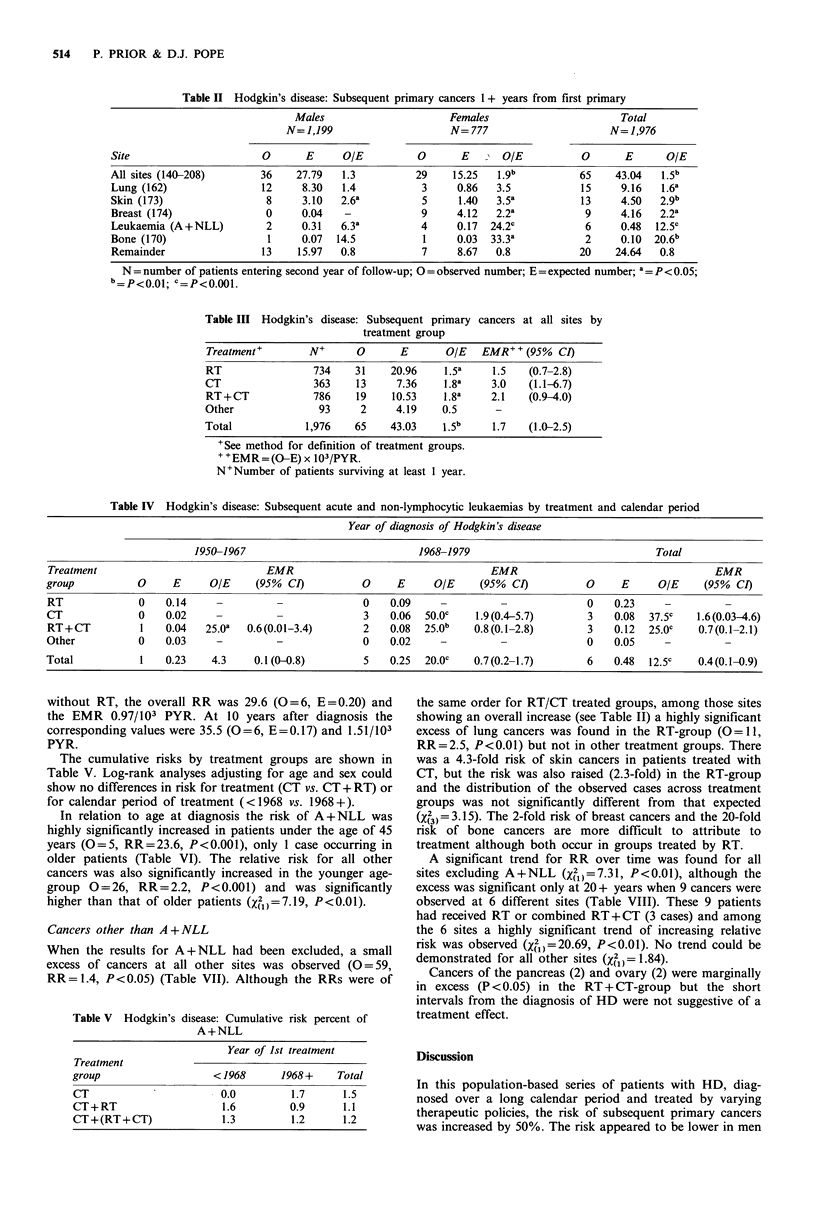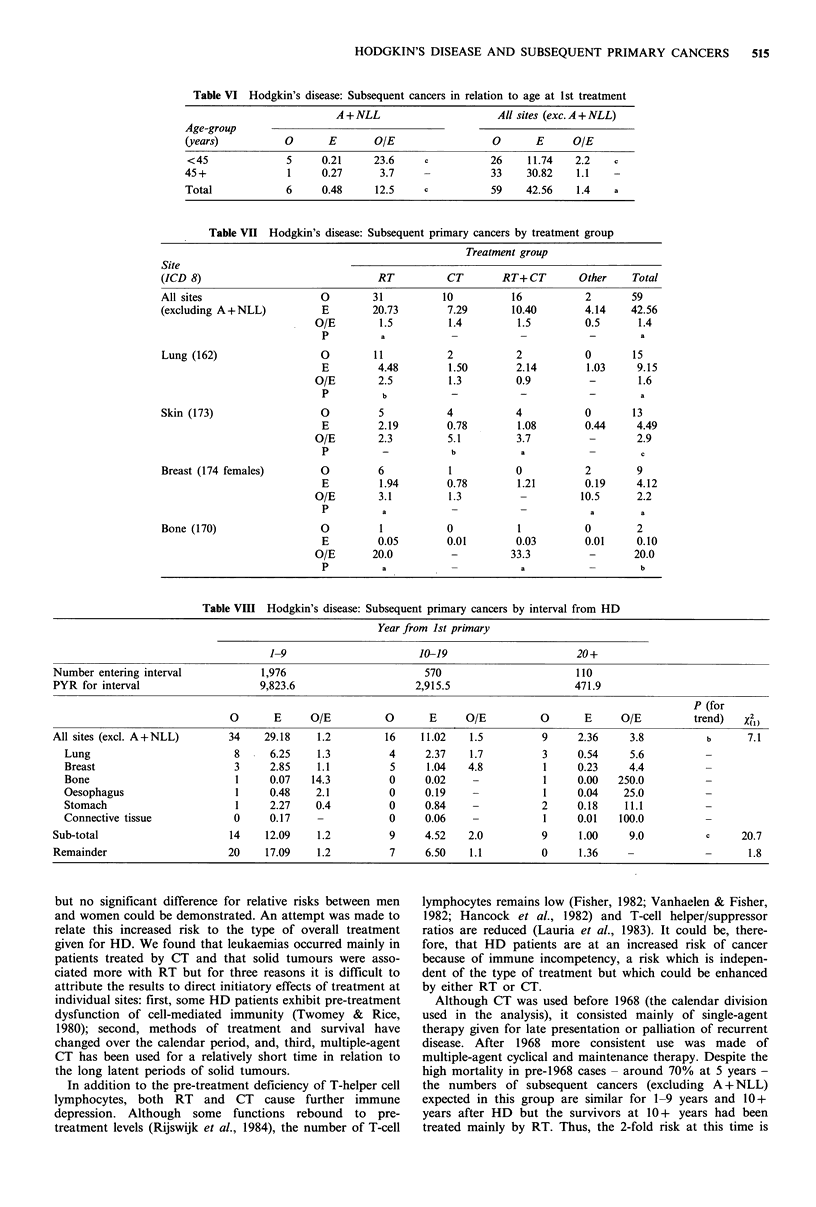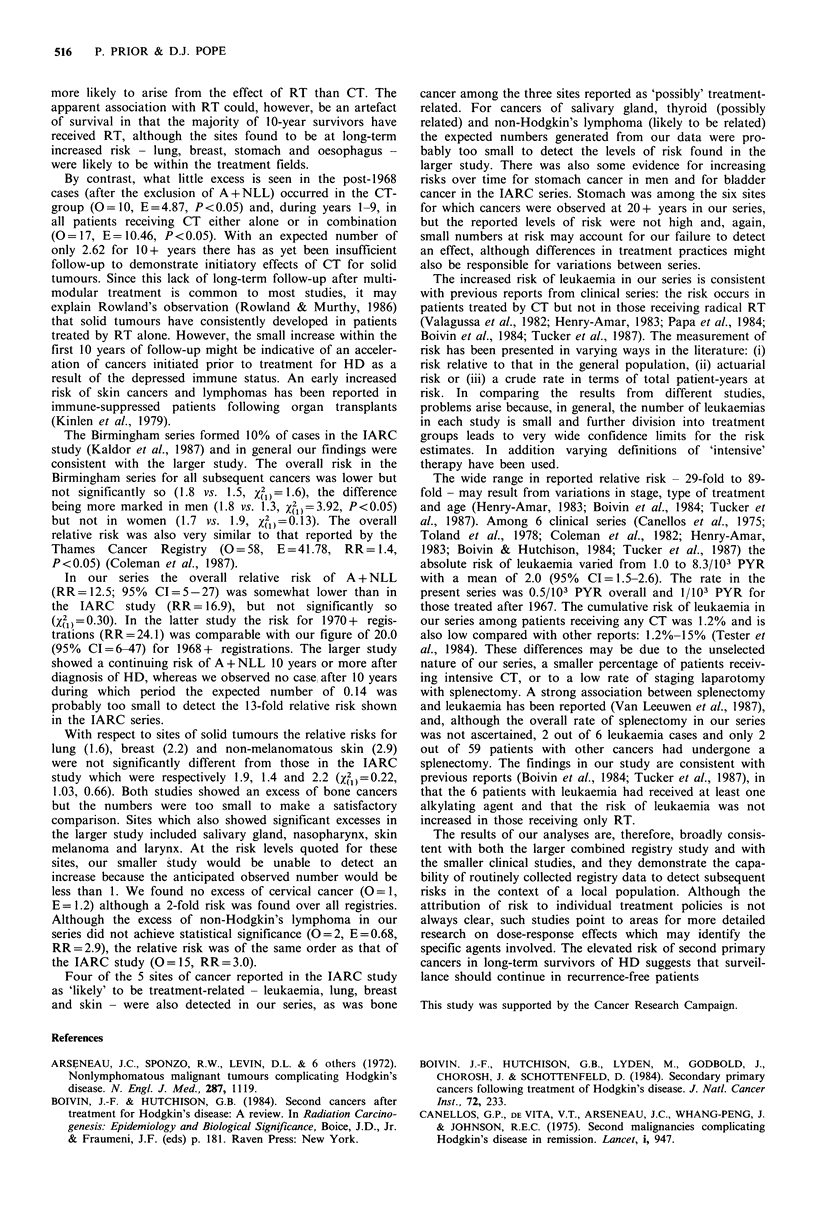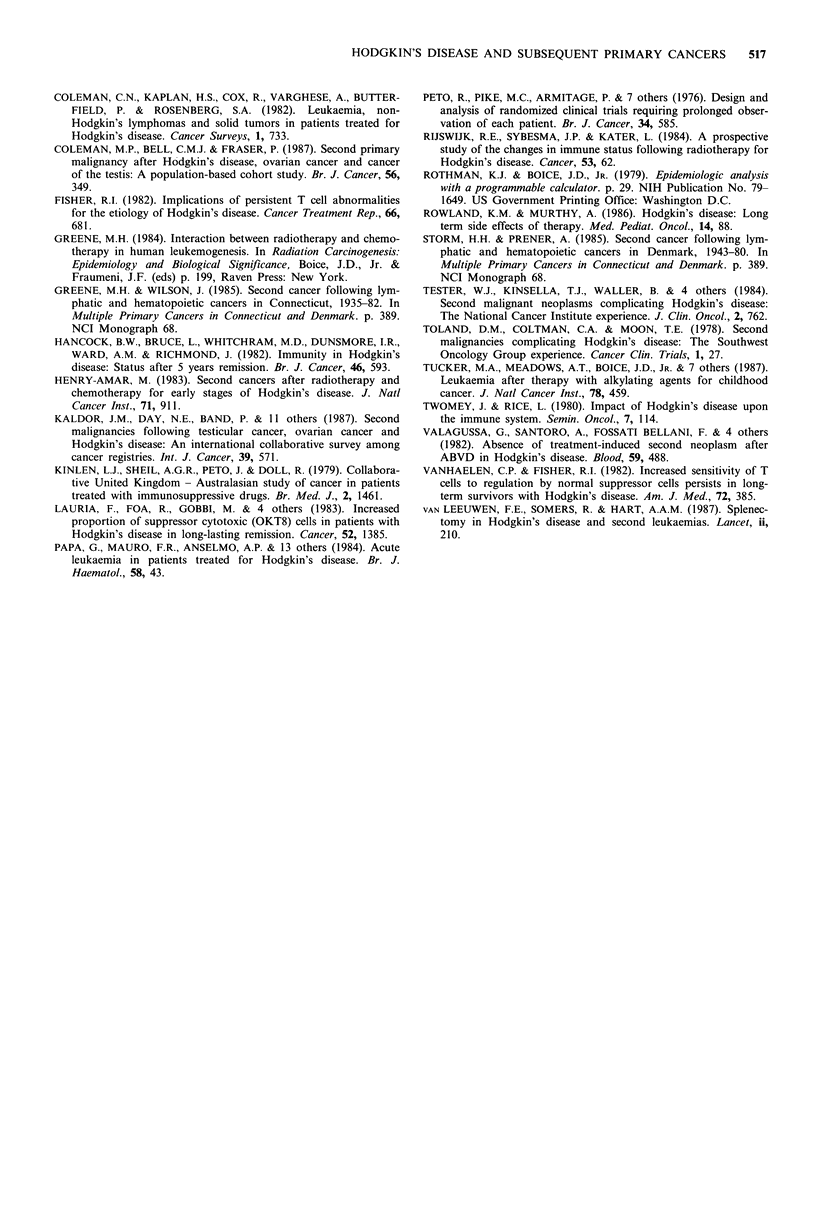# Hodgkin's disease: subsequent primary cancers in relation to treatment.

**DOI:** 10.1038/bjc.1988.253

**Published:** 1988-10

**Authors:** P. Prior, D. J. Pope

**Affiliations:** Department of Social Medicine, University of Birmingham, UK.

## Abstract

A consecutive series of 2,999 patients, diagnosed with Hodgkin's disease (HD) between 1950 and 1979, was assembled from the records of the Birmingham and West Midlands Cancer Registry and followed to the end of 1984. Cohort analyses of subsequent primary cancers among 1,976 patients, surviving one or more years (mean follow-up 6.7 person-years), were carried out in relation to overall treatment by radiotherapy (RT), chemotherapy (CT) or both modalities (CT + RT). Over all sites a 50% increase in risk, relative to the West Midlands population, was found [observed (O) = 65; relative risk (RR) = 1.5; P less than 0.01]. Among patients treated by CT (with or without RT) a significant increase in acute and non-lymphocytic leukaemias was found (O = 6; RR = 30.0; P less than 0.001). The excess risk was of the order of 1 per 1000 patient-years and the cumulative risk was 1.2%. Among solid tumours increased risks, which might be attributable to RT, occurred in the lung (O = 15; RR = 1.6; P less than 0.05), breast (O = 9; RR = 2.2; P less than 0.05) and bone (O = 2; RR = 20.0; P less than 0.01). The excess of skin cancers (O = 13; RR = 2.9; P less than 0.01) occurred mainly within 10 years of treatment with CT. The follow-up period is still insufficient to determine the long-term effect on the incidence of solid tumours with long latent periods from multiple-agent CT which became more frequently used in the early 1970s. A sub-set of these data was analysed over all treatments and the results were contributed to an international study co-ordinated by the International Agency for Research on Cancer, Lyon.


*Br. J. Cancer* (1988), **58**, 512–517 © The Macmillan Press Ltd., 1988

### Hodgkin's disease: subsequent primary cancers in relation to treatment

P. Prior & D.J. Pope

*Cancer Epidemiology Research Unit, Department of Social Medicine, University of Birmingham, Birmingham, B15 2TJ, UK.*

**Summary** A consecutive series of 2,999 patients, diagnosed with Hodgkin's disease (HD) between 1950 and 1979, was assembled from the records of the Birmingham and West Midlands Cancer Registry and followed to the end of 1984. Cohort analyses of subsequent primary cancers among 1,976 patients, surviving one or more years (mean follow-up 6.7 person-years), were carried out in relation to overall treatment by radiotherapy (RT), chemotherapy (CT) or both modalities (CT+RT). Over all sites a 50% increase in risk, relative to the West Midlands population, was found [observed (O)=65; relative risk (RR)=1.5; $P<0.01$]. Among patients treated by CT (with or without RT) a significant increase in acute and non-lymphocytic leukaemias was found (O=6; RR=30.0; $P<0.001$). The excess risk was of the order of 1 per 1000 patient-years and the cumulative risk was 1.2%. Among solid tumours increased risks, which might be attributable to RT, occurred in the lung (O=15; RR=1.6; $P<0.05$), breast (O=9; RR=2.2; $P<0.05$) and bone (O=2; RR=20.0; $P<0.01$). The excess of skin cancers (0=13; RR=2.9; $P<0.01$) occurred mainly within 10 years of treatment with CT. The follow-up period is still insufficient to determine the long-term effect on the incidence of solid tumours with long latent periods from multiple-agent CT which became more frequently used in the early 1970s. A sub-set of these data was analysed over all treatments and the results were contributed to an international study co-ordinated by the International Agency for Research on Cancer, Lyon.

Although recent CT regimes have improved the survival rate in HD, the longer period of remission, together with the carcinogenic nature of the drugs used to achieve it, have increased the risk of subsequent primary cancers in those who respond to treatment.

An early study on second primary cancers in patients treated for HD between 1953 and 1971 reported a 3-fold increase in solid tumours. No case of leukaemia was observed (Arseneau *et al.*, 1972). In the 1970s the use of combined treatments of RT and CT and of regimens of CT became more common and by the mid-1980s 29 cases of acute non-lymphocytic leukaemias developing after treatment for HD were cited (Greene, 1984). In the same year Boivin (1984) reviewed 11 studies reporting quantitative estimates of second primary risks at all sites. The estimates were based on relative risks in relation to expected risks in the general population. In a general review of the long-term effects of therapy in HD (Rowland & Murthy, 1986), the 10-year actuarial risk of subsequent leukaemias was given as 6–9% on the basis of 14 reports in the literature, with that for solid tumours ranging from 2–6%. Increased risks of solid tumours were not usually found in series with a median follow-up time of less than 7 years.

Although detailed information on the mode of treatment was available for the majority of these reports, the numbers of patients available for comparison have been very small and this has prevented the clear separation of the effects of different modalities of treatment on subsequent cancer risk. By contrast, population-based cancer registries can provide greater numbers for analysis but are not always able to distinguish treatment groups.

Among more than 3,000 patients with HD registered between 1935 and 1982 in Connecticut (Greene & Wilson, 1985) only 97 second primaries were observed, 2 of which were acute non-lymphocytic leukaemias. However, among solid tumours, increased risks were observed for buccal cavity, pharynx and respiratory system in men, and breast and thyroid in women. The overall risk in the Danish Cancer Registry material for 1943–1980 was similar to that of Connecticut but a highly significant excess of acute and non-lymphocytic leukaemia was observed. Among solid tumours only respiratory system in men and the bladder in women appeared to be at increased risk (Storm & Prener, 1985). Neither registry was able to identify groups treated by CT, but most of the long-term follow-up refers to patients treated by RT. A collaborative study, of material from 11 population-based cancer registries co-ordinated at the International Agency for Research on Cancer (IARC), was undertaken to increase the numbers of patients and second primaries for analysis (Kaldor *et al.*, 1987). The change in risk over calendar period was examined in order to test the effect of the introduction of multiple-agent CT. The overall risk (1.7-fold) was similar to the results from Connecticut and Denmark. A highly significant excess of acute and non-lymphocytic leukaemias was observed. The risk increased with calendar year of diagnosis of HD and remained at a high level for up to 15 years after treatment.

The present paper reports a follow-up study of all patients registered at the Birmingham and West Midlands Regional Cancer Registry between 1950 and 1979 and examines the incidence of subsequent primary cancers in terms of treatment, interval from first primary and calendar period. The type of treatment received is routinely recorded at registration and at follow-up. A sub-set of the data was analysed and the results were included in the IARC study and comprised 1950–1976 registrations with follow-up to the end of 1982. In the present analysis registrations for 1977–1979 have been added and the follow-up for the whole series extended to the end of 1984.

### Materials and methods

A series of 2,999 patients with a diagnosis of HD between 1950 and 1979 was identified from the Registry records. The age-distribution for 1,826 men and 1,173 women is given in Table I.

Cancer incidence rates were computed from the Registry data by site, sex, age and calendar period for all sites and 42 individual sites standardised to the 8th. Revision of the International Classification of Diseases (ICD8). The numbers of subsequent cancers that might be expected to occur, on the basis of no excess risk in the series, were computed by applying the appropriate incidence rates to the sex-, age- and calendar period-specific PYR. Subsequent primary cancers were identified from the Registry data and by scrutiny of the medical reports at active follow-up.

Two summary indices were used to assess the risk of

Correspondence: P. Prior.
Received 10 February 1988; and in revised form, 22 June 1988.

subsequent cancer in the series: relative risk (RR) defined as the 'observed number/expected number', and an excess morbidity rate (EMR) defined as '(observed-expected number)/ PYR x $10^3$'. The former measures the risk in the series relative to that in the general population, the latter measures it on an absolute scale. Significance testing for individual RRs assumed that the observed numbers followed a Poisson distribution and exact Poisson probabilities were computed for a 1-tailed test. Confidence limits for RR and EMR were computed using Byar's approximation (Rothman & Boice, 1979). Estimates of the cumulative risk of second cancers ($1-\Sigma P$) and comparisons between sub-groups were made by life-table and log-rank analyses using the methods of Peto *et al.* (1976).

Since the main purpose of the study was to examine the possible effects of treatment on subsequent cancer incidence, the following tabulations have been restricted to events occurring 1 or more years after the diagnosis of HD. Thus, cancers diagnosed at the same time or shortly after HD are excluded from the analyses.

In the analyses of the leukaemias, A+NLL refers to all cell-types specified as 'acute' and all other types specified as 'non-lymphocytic', i.e. other leukaemias of the myeloid series.

In general the numbers of second cancers at specific sites were too small to allow fine division of the data by type and intensity of treatment or by interval between different types of treatment. After a preliminary analysis in terms of type of first treatment and taking into consideration the varying patterns of order of treatments over the time-period, it was decided that a more meaningful division could be based on overall treatment defined in relation to RT and CT. Four groups were defined as follows:

1. RT: radiotherapy only
2. CT: chemotherapy only
3. RT+CT: radiotherapy and chemotherapy – either concurrent or at intervals
4. OTHER: not covered by 1–3, i.e. not treated, surgery only, surgery and hormone, hormone only.

Patients in groups 1–3 may also have been treated surgically with/without hormones.

### Results

*Follow-up*

Of the 2,999 patients in the series, 1,023 (34.1%) died within the first year. Among 1950–1967 registrations, 38.5% died within 1 year and 70.4% survived less than 5 years. For registrations after 1967, the figures were 30.4% ,and 50.8% respectively. By the end of the survey 74.2% of all patients had died; 0.7% were incompletely traced and 0.4% had emigrated. The total series was observed for 15,506 PYR (mean=5.2 PYR) and 1-year survivors for 15,187 PYR (mean=7.7 PYR).

*Treatment groups*

In the total series 936 patients had received RT of which

**Table I** Hodgkin's disease: Distribution of all patients by age at first treatment

| Age (years) | Males Number | (%) | Females Number | (%) | Total |
|---|---|---|---|---|---|
| <15 | 80 | | 51 | | 131 (4.4) |
| 15–29 | 481 | | 332 | | 813 (17.1) |
| 30–44 | 413 | | 223 | | 636 (21.2) |
| 45–59 | 447 | | 214 | | 661 (22.0) |
| 60–74 | 334 | | 257 | | 591 (19.7) |
| 70+ | 71 | | 96 | | 167 (5.6) |
| Total | 1,826 | (60.9) | 1,173 | (39.1) | 2,999 (100.0) |

36.2% were also treated surgically and 7.1% received hormones; 652 received CT (surgery=20.1%, hormone=59.8%); 947 received CT and RT (surgery=25.2%, hormone 59.5%). Among the remaining 464, 21.9% were treated by surgery and 7.5% received hormones. Of the 93 patients in this group who survived more than 1 year, 56 (60%) had been treated surgically (1 with additional hormone), 4 (4.3%) with hormone only. No treatment was given in 7 cases and for 26 patients the treatment was unknown.

*Cancer morbidity – all sites*

Among the total series 115 other primary cancers were diagnosed, of which 42 occurred previous to or coincidentally with HD. A further 8 second primaries were diagnosed in the first year, leaving 65 as the total of observed cases for analysis in 1,976 patients surviving at least 1 year for an expectation of 43.04 (RR=1.5, $P<0.01$), over all treatment groups (Table II). Although the RRs for men and women were not significantly different ($\chi^2_{(1)}=2.01$), the additional excess in women was due mainly to the 2-fold increase in breast cancers (O=9, RR=2.02; $P<0.05$). A moderate excess of non-melanomatous skin cancers was observed in both men and women. No case of melanoma of skin occurred. Six cases of A+NLL were diagnosed and the risk was significantly increased in both men and women (O=6, RR=12.5, $P<0.001$). The combined results for men and women showed a highly significant excess of bone cancers (O=2, RR=20.6; $P<0.01$) and a moderate excess of lung cancers (O=15, RR=1.6; $P<0.05$). The remainders for other sites showed relative risks close to 1.0 and no individual site showed a significant deficit of second primaries.

For the 3 groups treated by RT or CT, the risk at all sites was significantly increased to a similar extent. No excess occurred in the remaining patients (Table III).

*Acute and non-lymphocytic leukaemia*

No case of chronic lymphatic leukaemia was observed (E=0.27). For an expectation of 0.48, 6 cases of A+NLL were diagnosed between 4 and 10 years after the first treatment for HD. The histologically confirmed cell-types were acute myeloid (3), acute monocytic (2) and erythroleukaemia (1). The results by treatment group and calendar period are shown in Table IV. The one patient developing a leukaemia before 1968 had received RT with cyclophosphamide and vinblastine as single agents. Acute myeloid leukaemia was confirmed 10 years later. The remaining 5 cases of leukaemia arose in patients treated after 1968, all of whom had received multiple-agent CT: 4 patients received nitrogen mustard, procarbazine and vinblastine (MVPP), 2 of these received additional cyclophosphamide and a third later RT and vincristine. The fifth patient was treated initially with RT and vinblastine followed by combination CT consisting of cyclophosphamide, nitrogen mustard and procarbazine. All 5 patients had received hormone treatment, either independently or as part of a polychemotherapeutic regimen.

Patients treated after 1967 experienced a 20-fold risk (O=5, E=0.25, RR=20.0, 95% CI 6.4–46.7) of A+NLL, equivalent in round terms to an EMR of 1 in 1000 PYR (95% CI 0.2–1.7). No case of A+NLL occurred in the 'RT' or 'other' treatment groups, although the expected number (0.11) was comparable with that of the 'CT' and 'CT+RT' combined (0.14). The apparent differences between CT and CT+RT for both RR and EMR were not significant. However, there is no evidence in these results to suggest that combined modality treatment (CT+RT) incurs a greater risk of A+NLL than CT alone.

In relation to time since diagnosis of HD, the RR of A+NLL for the total series was 17.5 (O=6, E=0.34) 10 years after first treatment and 12.5 (O=6, E=0.48) overall. For patients who had received CT at any time, with or

**Table II** Hodgkin's disease: Subsequent primary cancers 1+ years from first primary

| | *Males* $N=1,199$ | | | *Females* $N=777$ | | | *Total* $N=1,976$ | | |
|---|---|---|---|---|---|---|---|---|---|
| *Site* | *O* | *E* | *O/E* | *O* | *E* | *O/E* | *O* | *E* | *O/E* |
| All sites (140–208) | 36 | 27.79 | 1.3 | 29 | 15.25 | 1.9[b] | 65 | 43.04 | 1.5[b] |
| Lung (162) | 12 | 8.30 | 1.4 | 3 | 0.86 | 3.5 | 15 | 9.16 | 1.6[a] |
| Skin (173) | 8 | 3.10 | 2.6[a] | 5 | 1.40 | 3.5[a] | 13 | 4.50 | 2.9[b] |
| Breast (174) | 0 | 0.04 | – | 9 | 4.12 | 2.2[a] | 9 | 4.16 | 2.2[a] |
| Leukaemia (A+NLL) | 2 | 0.31 | 6.3[a] | 4 | 0.17 | 24.2[c] | 6 | 0.48 | 12.5[c] |
| Bone (170) | 1 | 0.07 | 14.5 | 1 | 0.03 | 33.3[a] | 2 | 0.10 | 20.6[b] |
| Remainder | 13 | 15.97 | 0.8 | 7 | 8.67 | 0.8 | 20 | 24.64 | 0.8 |

N = number of patients entering second year of follow-up; O = observed number; E = expected number; [a] = $P<0.05$; [b] = $P<0.01$; [c] = $P<0.001$.

**Table III** Hodgkin's disease: Subsequent primary cancers at all sites by treatment group

| *Treatment*[+] | $N^+$ | *O* | *E* | *O/E* | *EMR*[++] *(95% CI)* |
|---|---|---|---|---|---|
| RT | 734 | 31 | 20.96 | 1.5[a] | 1.5 (0.7–2.8) |
| CT | 363 | 13 | 7.36 | 1.8[a] | 3.0 (1.1–6.7) |
| RT+CT | 786 | 19 | 10.53 | 1.8[a] | 2.1 (0.9–4.0) |
| Other | 93 | 2 | 4.19 | 0.5 | – |
| Total | 1,976 | 65 | 43.03 | 1.5[b] | 1.7 (1.0–2.5) |

[+]See method for definition of treatment groups.
[++]EMR = (O–E) × $10^3$/PYR.
$N^+$Number of patients surviving at least 1 year.

**Table IV** Hodgkin's disease: Subsequent acute and non-lymphocytic leukaemias by treatment and calendar period

| | *Year of diagnosis of Hodgkin's disease* | | | | | | | | | | | |
|---|---|---|---|---|---|---|---|---|---|---|---|---|
| | *1950–1967* | | | | *1968–1979* | | | | *Total* | | | |
| *Treatment group* | *O* | *E* | *O/E* | *EMR (95% CI)* | *O* | *E* | *O/E* | *EMR (95% CI)* | *O* | *E* | *O/E* | *EMR (95% CI)* |
| RT | 0 | 0.14 | – | – | 0 | 0.09 | – | – | 0 | 0.23 | – | – |
| CT | 0 | 0.02 | – | – | 3 | 0.06 | 50.0[c] | 1.9 (0.4–5.7) | 3 | 0.08 | 37.5[c] | 1.6 (0.03–4.6) |
| RT+CT | 1 | 0.04 | 25.0[a] | 0.6 (0.01–3.4) | 2 | 0.08 | 25.0[b] | 0.8 (0.1–2.8) | 3 | 0.12 | 25.0[c] | 0.7 (0.1–2.1) |
| Other | 0 | 0.03 | – | – | 0 | 0.02 | – | – | 0 | 0.05 | – | – |
| Total | 1 | 0.23 | 4.3 | 0.1 (0–0.8) | 5 | 0.25 | 20.0[c] | 0.7 (0.2–1.7) | 6 | 0.48 | 12.5[c] | 0.4 (0.1–0.9) |

without RT, the overall RR was 29.6 (O=6, E=0.20) and the EMR 0.97/$10^3$ PYR. At 10 years after diagnosis the corresponding values were 35.5 (O=6, E=0.17) and 1.51/$10^3$ PYR.

The cumulative risks by treatment groups are shown in Table V. Log-rank analyses adjusting for age and sex could show no differences in risk for treatment (CT *vs.* CT+RT) or for calendar period of treatment (<1968 *vs.* 1968+).

In relation to age at diagnosis the risk of A+NLL was highly significantly increased in patients under the age of 45 years (O=5, RR=23.6, $P<0.001$), only 1 case occurring in older patients (Table VI). The relative risk for all other cancers was also significantly increased in the younger age-group O=26, RR=2.2, $P<0.001$) and was significantly higher than that of older patients ($\chi^2_{(1)}=7.19$, $P<0.01$).

### *Cancers other than A+NLL*

When the results for A+NLL had been excluded, a small excess of cancers at all other sites was observed (O=59, RR=1.4, $P<0.05$) (Table VII). Although the RRs were of

**Table V** Hodgkin's disease: Cumulative risk percent of A+NLL

| | *Year of 1st treatment* | | |
|---|---|---|---|
| *Treatment group* | *<1968* | *1968+* | *Total* |
| CT | 0.0 | 1.7 | 1.5 |
| CT+RT | 1.6 | 0.9 | 1.1 |
| CT+(RT+CT) | 1.3 | 1.2 | 1.2 |

the same order for RT/CT treated groups, among those sites showing an overall increase (see Table II) a highly significant excess of lung cancers was found in the RT-group (O=11, RR=2.5, $P<0.01$) but not in other treatment groups. There was a 4.3-fold risk of skin cancers in patients treated with CT, but the risk was also raised (2.3-fold) in the RT-group and the distribution of the observed cases across treatment groups was not significantly different from that expected ($\chi^2_{(3)}=3.15$). The 2-fold risk of breast cancers and the 20-fold risk of bone cancers are more difficult to attribute to treatment although both occur in groups treated by RT.

A significant trend for RR over time was found for all sites excluding A+NLL ($\chi^2_{(1)}=7.31$, $P<0.01$), although the excess was significant only at 20+ years when 9 cancers were observed at 6 different sites (Table VIII). These 9 patients had received RT or combined RT+CT (3 cases) and among the 6 sites a highly significant trend of increasing relative risk was observed ($\chi^2_{(1)}=20.69$, $P<0.01$). No trend could be demonstrated for all other sites ($\chi^2_{(1)}=1.84$).

Cancers of the pancreas (2) and ovary (2) were marginally in excess ($P<0.05$) in the RT+CT-group but the short intervals from the diagnosis of HD were not suggestive of a treatment effect.

### Discussion

In this population-based series of patients with HD, diagnosed over a long calendar period and treated by varying therapeutic policies, the risk of subsequent primary cancers was increased by 50%. The risk appeared to be lower in men

**Table VI** Hodgkin's disease: Subsequent cancers in relation to age at 1st treatment

| Age-group (years) | *A+NLL* | | | | *All sites (exc. A+NLL)* | | | |
|---|---|---|---|---|---|---|---|---|
| | *O* | *E* | *O/E* | | *O* | *E* | *O/E* | |
| <45 | 5 | 0.21 | 23.6 | c | 26 | 11.74 | 2.2 | c |
| 45+ | 1 | 0.27 | 3.7 | – | 33 | 30.82 | 1.1 | – |
| Total | 6 | 0.48 | 12.5 | c | 59 | 42.56 | 1.4 | a |

**Table VII** Hodgkin's disease: Subsequent primary cancers by treatment group

| Site (ICD 8) | | *Treatment group* | | | | |
|---|---|---|---|---|---|---|
| | | *RT* | *CT* | *RT+CT* | *Other* | *Total* |
| All sites | O | 31 | 10 | 16 | 2 | 59 |
| (excluding A+NLL) | E | 20.73 | 7.29 | 10.40 | 4.14 | 42.56 |
| | O/E | 1.5 | 1.4 | 1.5 | 0.5 | 1.4 |
| | P | a | – | – | – | a |
| Lung (162) | O | 11 | 2 | 2 | 0 | 15 |
| | E | 4.48 | 1.50 | 2.14 | 1.03 | 9.15 |
| | O/E | 2.5 | 1.3 | 0.9 | – | 1.6 |
| | P | b | – | – | – | a |
| Skin (173) | O | 5 | 4 | 4 | 0 | 13 |
| | E | 2.19 | 0.78 | 1.08 | 0.44 | 4.49 |
| | O/E | 2.3 | 5.1 | 3.7 | – | 2.9 |
| | P | – | b | a | – | c |
| Breast (174 females) | O | 6 | 1 | 0 | 2 | 9 |
| | E | 1.94 | 0.78 | 1.21 | 0.19 | 4.12 |
| | O/E | 3.1 | 1.3 | – | 10.5 | 2.2 |
| | P | a | – | – | a | a |
| Bone (170) | O | 1 | 0 | 1 | 0 | 2 |
| | E | 0.05 | 0.01 | 0.03 | 0.01 | 0.10 |
| | O/E | 20.0 | – | 33.3 | – | 20.0 |
| | P | a | – | a | – | b |

**Table VIII** Hodgkin's disease: Subsequent primary cancers by interval from HD

| | *Year from 1st primary* | | | | | | | | | | |
|---|---|---|---|---|---|---|---|---|---|---|---|
| | *1–9* | | | *10–19* | | | *20+* | | | | |
| Number entering interval | 1,976 | | | 570 | | | 110 | | | | |
| PYR for interval | 9,823.6 | | | 2,915.5 | | | 471.9 | | | | |
| | O | E | O/E | O | E | O/E | O | E | O/E | P (for trend) | $\chi^2_{(1)}$ |
| All sites (excl. A+NLL) | 34 | 29.18 | 1.2 | 16 | 11.02 | 1.5 | 9 | 2.36 | 3.8 | b | 7.1 |
| Lung | 8 | 6.25 | 1.3 | 4 | 2.37 | 1.7 | 3 | 0.54 | 5.6 | – | |
| Breast | 3 | 2.85 | 1.1 | 5 | 1.04 | 4.8 | 1 | 0.23 | 4.4 | – | |
| Bone | 1 | 0.07 | 14.3 | 0 | 0.02 | – | 1 | 0.00 | 250.0 | – | |
| Oesophagus | 1 | 0.48 | 2.1 | 0 | 0.19 | – | 1 | 0.04 | 25.0 | – | |
| Stomach | 1 | 2.27 | 0.4 | 0 | 0.84 | – | 2 | 0.18 | 11.1 | – | |
| Connective tissue | 0 | 0.17 | – | 0 | 0.06 | – | 1 | 0.01 | 100.0 | – | |
| Sub-total | 14 | 12.09 | 1.2 | 9 | 4.52 | 2.0 | 9 | 1.00 | 9.0 | c | 20.7 |
| Remainder | 20 | 17.09 | 1.2 | 7 | 6.50 | 1.1 | 0 | 1.36 | – | – | 1.8 |

but no significant difference for relative risks between men and women could be demonstrated. An attempt was made to relate this increased risk to the type of overall treatment given for HD. We found that leukaemias occurred mainly in patients treated by CT and that solid tumours were associated more with RT but for three reasons it is difficult to attribute the results to direct initiatory effects of treatment at individual sites: first, some HD patients exhibit pre-treatment dysfunction of cell-mediated immunity (Twomey & Rice, 1980); second, methods of treatment and survival have changed over the calendar period, and, third, multiple-agent CT has been used for a relatively short time in relation to the long latent periods of solid tumours.

In addition to the pre-treatment deficiency of T-helper cell lymphocytes, both RT and CT cause further immune depression. Although some functions rebound to pre-treatment levels (Rijswijk *et al.*, 1984), the number of T-cell lymphocytes remains low (Fisher, 1982; Vanhaelen & Fisher, 1982; Hancock *et al.*, 1982) and T-cell helper/suppressor ratios are reduced (Lauria *et al.*, 1983). It could be, therefore, that HD patients are at an increased risk of cancer because of immune incompetency, a risk which is independent of the type of treatment but which could be enhanced by either RT or CT.

Although CT was used before 1968 (the calendar division used in the analysis), it consisted mainly of single-agent therapy given for late presentation or palliation of recurrent disease. After 1968 more consistent use was made of multiple-agent cyclical and maintenance therapy. Despite the high mortality in pre-1968 cases – around 70% at 5 years – the numbers of subsequent cancers (excluding A+NLL) expected in this group are similar for 1–9 years and 10+ years after HD but the survivors at 10+ years had been treated mainly by RT. Thus, the 2-fold risk at this time is more likely to arise from the effect of RT than CT. The apparent association with RT could, however, be an artefact of survival in that the majority of 10-year survivors have received RT, although the sites found to be at long-term increased risk – lung, breast, stomach and oesophagus – were likely to be within the treatment fields.

By contrast, what little excess is seen in the post-1968 cases (after the exclusion of A+NLL) occurred in the CT-group (O = 10, E = 4.87, $P<0.05$) and, during years 1–9, in all patients receiving CT either alone or in combination (O = 17, E = 10.46, $P<0.05$). With an expected number of only 2.62 for 10+ years there has as yet been insufficient follow-up to demonstrate initiatory effects of CT for solid tumours. Since this lack of long-term follow-up after multi-modular treatment is common to most studies, it may explain Rowland's observation (Rowland & Murthy, 1986) that solid tumours have consistently developed in patients treated by RT alone. However, the small increase within the first 10 years of follow-up might be indicative of an acceleration of cancers initiated prior to treatment for HD as a result of the depressed immune status. An early increased risk of skin cancers and lymphomas has been reported in immune-suppressed patients following organ transplants (Kinlen *et al.*, 1979).

The Birmingham series formed 10% of cases in the IARC study (Kaldor *et al.*, 1987) and in general our findings were consistent with the larger study. The overall risk in the Birmingham series for all subsequent cancers was lower but not significantly so (1.8 *vs.* 1.5, $\chi^2_{(1)}=1.6$), the difference being more marked in men (1.8 *vs.* 1.3, $\chi^2_{(1)}=3.92$, $P<0.05$) but not in women (1.7 *vs.* 1.9, $\chi^2_{(1)}=0.13$). The overall relative risk was also very similar to that reported by the Thames Cancer Registry (O = 58, E = 41.78, RR = 1.4, $P<0.05$) (Coleman *et al.*, 1987).

In our series the overall relative risk of A+NLL (RR = 12.5; 95% CI = 5 – 27) was somewhat lower than in the IARC study (RR = 16.9), but not significantly so ($\chi^2_{(1)}=0.30$). In the latter study the risk for 1970+ registrations (RR = 24.1) was comparable with our figure of 20.0 (95% CI = 6–47) for 1968+ registrations. The larger study showed a continuing risk of A+NLL 10 years or more after diagnosis of HD, whereas we observed no case after 10 years during which period the expected number of 0.14 was probably too small to detect the 13-fold relative risk shown in the IARC series.

With respect to sites of solid tumours the relative risks for lung (1.6), breast (2.2) and non-melanomatous skin (2.9) were not significantly different from those in the IARC study which were respectively 1.9, 1.4 and 2.2 ($\chi^2_{(1)}=0.22$, 1.03, 0.66). Both studies showed an excess of bone cancers but the numbers were too small to make a satisfactory comparison. Sites which also showed significant excesses in the larger study included salivary gland, nasopharynx, skin melanoma and larynx. At the risk levels quoted for these sites, our smaller study would be unable to detect an increase because the anticipated observed number would be less than 1. We found no excess of cervical cancer (O = 1, E = 1.2) although a 2-fold risk was found over all registries. Although the excess of non-Hodgkin's lymphoma in our series did not achieve statistical significance (O = 2, E = 0.68, RR = 2.9), the relative risk was of the same order as that of the IARC study (O = 15, RR = 3.0).

Four of the 5 sites of cancer reported in the IARC study as 'likely' to be treatment-related – leukaemia, lung, breast and skin – were also detected in our series, as was bone cancer among the three sites reported as 'possibly' treatment-related. For cancers of salivary gland, thyroid (possibly related) and non-Hodgkin's lymphoma (likely to be related) the expected numbers generated from our data were probably too small to detect the levels of risk found in the larger study. There was also some evidence for increasing risks over time for stomach cancer in men and for bladder cancer in the IARC series. Stomach was among the six sites for which cancers were observed at 20+ years in our series, but the reported levels of risk were not high and, again, small numbers at risk may account for our failure to detect an effect, although differences in treatment practices might also be responsible for variations between series.

The increased risk of leukaemia in our series is consistent with previous reports from clinical series: the risk occurs in patients treated by CT but not in those receiving radical RT (Valagussa *et al.*, 1982; Henry-Amar, 1983; Papa *et al.*, 1984; Boivin *et al.*, 1984; Tucker *et al.*, 1987). The measurement of risk has been presented in varying ways in the literature: (i) risk relative to that in the general population, (ii) actuarial risk or (iii) a crude rate in terms of total patient-years at risk. In comparing the results from different studies, problems arise because, in general, the number of leukaemias in each study is small and further division into treatment groups leads to very wide confidence limits for the risk estimates. In addition varying definitions of 'intensive' therapy have been used.

The wide range in reported relative risk – 29-fold to 89-fold – may result from variations in stage, type of treatment and age (Henry-Amar, 1983; Boivin *et al.*, 1984; Tucker *et al.*, 1987). Among 6 clinical series (Canellos *et al.*, 1975; Toland *et al.*, 1978; Coleman *et al.*, 1982; Henry-Amar, 1983; Boivin & Hutchison, 1984; Tucker *et al.*, 1987) the absolute risk of leukaemia varied from 1.0 to $8.3/10^3$ PYR with a mean of 2.0 (95% CI = 1.5–2.6). The rate in the present series was $0.5/10^3$ PYR overall and $1/10^3$ PYR for those treated after 1967. The cumulative risk of leukaemia in our series among patients receiving any CT was 1.2% and is also low compared with other reports: 1.2%–15% (Tester *et al.*, 1984). These differences may be due to the unselected nature of our series, a smaller percentage of patients receiving intensive CT, or to a low rate of staging laparotomy with splenectomy. A strong association between splenectomy and leukaemia has been reported (Van Leeuwen *et al.*, 1987), and, although the overall rate of splenectomy in our series was not ascertained, 2 out of 6 leukaemia cases and only 2 out of 59 patients with other cancers had undergone a splenectomy. The findings in our study are consistent with previous reports (Boivin *et al.*, 1984; Tucker *et al.*, 1987), in that the 6 patients with leukaemia had received at least one alkylating agent and that the risk of leukaemia was not increased in those receiving only RT.

The results of our analyses are, therefore, broadly consistent with both the larger combined registry study and with the smaller clinical studies, and they demonstrate the capability of routinely collected registry data to detect subsequent risks in the context of a local population. Although the attribution of risk to individual treatment policies is not always clear, such studies point to areas for more detailed research on dose-response effects which may identify the specific agents involved. The elevated risk of second primary cancers in long-term survivors of HD suggests that surveillance should continue in recurrence-free patients

This study was supported by the Cancer Research Campaign.

### References

ARSENEAU, J.C., SPONZO, R.W., LEVIN, D.L. & 6 others (1972). Nonlymphomatous malignant tumours complicating Hodgkin's disease. *N. Engl. J. Med.*, **287**, 1119.

BOIVIN, J.-F. & HUTCHISON, G.B. (1984). Second cancers after treatment for Hodgkin's disease: A review. In *Radiation Carcinogenesis: Epidemiology and Biological Significance*, Boice, J.D., Jr. & Fraumeni, J.F. (eds) p. 181. Raven Press: New York.

BOIVIN, J.-F., HUTCHISON, G.B., LYDEN, M., GODBOLD, J., CHOROSH, J. & SCHOTTENFELD, D. (1984). Secondary primary cancers following treatment of Hodgkin's disease. *J. Natl. Cancer Inst.*, **72**, 233.

CANELLOS, G.P., DE VITA, V.T., ARSENEAU, J.C., WHANG-PENG, J. & JOHNSON, R.E.C. (1975). Second malignancies complicating Hodgkin's disease in remission. *Lancet*, **i**, 947.

COLEMAN, C.N., KAPLAN, H.S., COX, R., VARGHESE, A., BUTTERFIELD, P. & ROSENBERG, S.A. (1982). Leukaemia, non-Hodgkin's lymphomas and solid tumors in patients treated for Hodgkin's disease. *Cancer Surveys*, **1**, 733.

COLEMAN, M.P., BELL, C.M.J. & FRASER, P. (1987). Second primary malignancy after Hodgkin's disease, ovarian cancer and cancer of the testis: A population-based cohort study. *Br. J. Cancer*, **56**, 349.

FISHER, R.I. (1982). Implications of persistent T cell abnormalities for the etiology of Hodgkin's disease. *Cancer Treatment Rep.*, **66**, 681.

GREENE, M.H. (1984). Interaction between radiotherapy and chemotherapy in human leukemogenesis. In *Radiation Carcinogenesis: Epidemiology and Biological Significance,* Boice, J.D., Jr. & Fraumeni, J.F. (eds) p. 199, Raven Press: New York.

GREENE, M.H. & WILSON, J. (1985). Second cancer following lymphatic and hematopoietic cancers in Connecticut, 1935–82. In *Multiple Primary Cancers in Connecticut and Denmark*. p. 389. NCI Monograph 68.

HANCOCK, B.W., BRUCE, L., WHITCHRAM, M.D., DUNSMORE, I.R., WARD, A.M. & RICHMOND, J. (1982). Immunity in Hodgkin's disease: Status after 5 years remission. *Br. J. Cancer*, **46**, 593.

HENRY-AMAR, M. (1983). Second cancers after radiotherapy and chemotherapy for early stages of Hodgkin's disease. *J. Natl Cancer Inst.*, **71**, 911.

KALDOR, J.M., DAY, N.E., BAND, P. & 11 others (1987). Second malignancies following testicular cancer, ovarian cancer and Hodgkin's disease: An international collaborative survey among cancer registries. *Int. J. Cancer*, **39**, 571.

KINLEN, L.J., SHEIL, A.G.R., PETO, J. & DOLL, R. (1979). Collaborative United Kingdom – Australasian study of cancer in patients treated with immunosuppressive drugs. *Br. Med. J.*, **2**, 1461.

LAURIA, F., FOA, R., GOBBI, M. & 4 others (1983). Increased proportion of suppressor cytotoxic (OKT8) cells in patients with Hodgkin's disease in long-lasting remission. *Cancer*, **52**, 1385.

PAPA, G., MAURO, F.R., ANSELMO, A.P. & 13 others (1984). Acute leukaemia in patients treated for Hodgkin's disease. *Br. J. Haematol.*, **58**, 43.

PETO, R., PIKE, M.C., ARMITAGE, P. & 7 others (1976). Design and analysis of randomized clinical trials requiring prolonged observation of each patient. *Br. J. Cancer*, **34**, 585.

RIJSWIJK, R.E., SYBESMA, J.P. & KATER, L. (1984). A prospective study of the changes in immune status following radiotherapy for Hodgkin's disease. *Cancer*, **53**, 62.

ROTHMAN, K.J. & BOICE, J.D., JR. (1979). *Epidemiologic analysis with a programmable calculator*. p. 29. NIH Publication No. 79-1649. US Government Printing Office: Washington D.C.

ROWLAND, K.M. & MURTHY, A. (1986). Hodgkin's disease: Long term side effects of therapy. *Med. Pediat. Oncol.*, **14**, 88.

STORM, H.H. & PRENER, A. (1985). Second cancer following lymphatic and hematopoietic cancers in Denmark, 1943–80. In *Multiple Primary Cancers in Connecticut and Denmark*. p. 389. NCI Monograph 68.

TESTER, W.J., KINSELLA, T.J., WALLER, B. & 4 others (1984). Second malignant neoplasms complicating Hodgkin's disease: The National Cancer Institute experience. *J. Clin. Oncol.*, **2**, 762.

TOLAND, D.M., COLTMAN, C.A. & MOON, T.E. (1978). Second malignancies complicating Hodgkin's disease: The Southwest Oncology Group experience. *Cancer Clin. Trials*, **1**, 27.

TUCKER, M.A., MEADOWS, A.T., BOICE, J.D., JR. & 7 others (1987). Leukaemia after therapy with alkylating agents for childhood cancer. *J. Natl Cancer Inst.*, **78**, 459.

TWOMEY, J. & RICE, L. (1980). Impact of Hodgkin's disease upon the immune system. *Semin. Oncol.*, **7**, 114.

VALAGUSSA, G., SANTORO, A., FOSSATI BELLANI, F. & 4 others (1982). Absence of treatment-induced second neoplasm after ABVD in Hodgkin's disease. *Blood*, **59**, 488.

VANHAELEN, C.P. & FISHER, R.I. (1982). Increased sensitivity of T cells to regulation by normal suppressor cells persists in long-term survivors with Hodgkin's disease. *Am. J. Med.*, **72**, 385.

VAN LEEUWEN, F.E., SOMERS, R. & HART, A.A.M. (1987). Splenectomy in Hodgkin's disease and second leukaemias. *Lancet*, **ii**, 210.